# Ventriculoperitoneal shunt for tuberculous meningitis-associated hydrocephalus: long-term outcomes and complications

**DOI:** 10.1186/s12879-023-08661-7

**Published:** 2023-10-30

**Authors:** Xiao Zhang, Pengtao Li, Junxian Wen, Jianbo Chang, Yihao Chen, Rui Yin, Houshi Xu, Xiaoyu Liu, Lang Yang, Junji Wei

**Affiliations:** Department of Neurosurgery, Peking Union Medical College Hospital, Peking Union Medical College, Chinese Academy of Medical Sciences, Beijing, China

**Keywords:** Ventriculoperitoneal Shunt, Tuberculous meningitis, Hydrocephalus, Complications

## Abstract

**Background:**

Hydrocephalus is a frequent complication of tuberculous meningitis (TBM), and ventriculoperitoneal shunt (VPS) has been shown to improve short-term prognosis for patients with TBM-associated hydrocephalus. However, questions remain about long-term prognosis and shunt-related complications. This study aims to provide a comprehensive assessment of both long-term prognosis and shunt-related complications in patients with TBM-induced hydrocephalus who have undergone VPS treatment.

**Methods:**

This retrospective study analyzed the clinical data of TBM patients with hydrocephalus treated with VPS at Peking Union Medical College Hospital between December 1999 and February 2023. Both short-term outcomes at discharge and long-term outcomes during follow-up were examined. Prognosis and shunt-related complications were assessed using the modified Rankin Scale (mRS) and the Activity of Daily Living (ADL) score to evaluate neurological function and autonomic living ability, respectively.

**Results:**

A total of 14 patients with TBM-associated hydrocephalus were included in this study. Of these, 92.9% (13/14) exhibited favorable short-term outcomes, while 57.1% (8/14) showed positive long-term outcomes. Initial results indicated 6 complete recoveries (CR), 7 partial recoveries (PR), and 1 treatment failure. No catheter-related complications were observed initially. Long-term results included 4 CRs, 4 PRs, and 6 treatment failures. A variety of shunt surgery-related complications were noted, including three instances of catheter obstruction, one of incision infection, one of catheter-related infection, one of acute cerebral infarction, and one of transient peritoneal irritation accompanied by diarrhea.

**Conclusions:**

VPS appears to be an effective and well-tolerated treatment for TBM-associated hydrocephalus, efficiently alleviating acute intracranial hypertension. Nonetheless, continuous long-term monitoring and proactive management are essential to mitigate the risk of catheter-related complications.

## Introduction

Tuberculous meningitis (TBM) is a severe form of meningitis induced by *Mycobacterium tuberculosis*, comprising approximately 1–5% of all tuberculosis cases [1, 2]. Globally, it’s estimated that at least 100,000 cases of TBM occur each year [2]. TBM is characterized by high rates of mortality and disability [3, 4]. Its early symptoms are often nonspecific; classic signs of meningitis, such as headache, fever, vomiting, and neck stiffness, tend to manifest progressively as the disease advances [5]. Without appropriate treatment, symptoms intensify, leading to elevated intracranial pressure, altered consciousness, focal neurological deficits, and even life-threatening brain herniation.

Hydrocephalus is the most frequent complication of TBM. Reports indicate that as many as 80% of TBM patients show signs of ventricular enlargement in the early stages, with hydrocephalus serving as a critical prognostic factor [6, 7]. Types of TBM-associated hydrocephalus include communicating, obstructive, and mixed. Medical treatments often combine anti-tubercular therapy with steroids and various diuretics like acetazolamide, methazolamide, furosemide, and mannitol. Nevertheless, in instances of acute intracranial pressure elevation stemming from acute-phase TBM, pharmacological interventions may not provide rapid relief, making surgical shunting a viable option for such patients.

Ventriculoperitoneal shunt (VPS) has demonstrated effectiveness in treating hydrocephalus related to TBM. For example, Raman et al. [8] recommended that patients with favorable Palur grading and TBM-associated hydrocephalus should undergo VPS as early as possible to significantly improve prognosis. Peng et al. [9] suggested that even Grade IV TBM patients could benefit substantially from VPS. However, this surgical procedure is not without risks, including shunt-related infections, blockages, over-drainage, and the potential for abdominal dissemination of tubercle bacilli [10]. Currently, there is a paucity of long-term follow-up studies addressing shunt-related complications and outcomes post-VPS for TBM-associated hydrocephalus. Thus, we conducted a single-center retrospective study with extended follow-up to thoroughly assess the efficacy and safety of VPS in patients with TBM-induced hydrocephalus.

## Methods

### Study design

This retrospective study enrolled patients with TBM-associated hydrocephalus who underwent VPS treatment at Peking Union Medical College Hospital from December 1999 to February 2023. Medical data, including baseline demographics, medical history, laboratory and imaging results, and follow-up outcomes were extracted from the hospital’s electronic medical record system. The study was approved by the Ethics Committee of Peking Union Medical College Hospital (HS-1571).

### TBM clinical diagnostic criteria

Clinical signs suggestive of meningitis included symptoms such as headache, vomiting, fever, neck stiffness, seizures, focal neurological deficits, or altered sensorium. Patients were categorized into one of three groups: ‘definite,‘ ‘probable,‘ or ‘possible’ TBM [11]. A diagnosis of ‘definite’ TBM was confirmed by the detection of acid-fast bacilli in cerebrospinal fluid (CSF) smears, the isolation of mycobacteria from CSF cultures, or positive nucleic acid amplification tests for tuberculosis. ‘Probable’ TBM was defined by a clinical assessment score of 10 or higher (or 12 or more if imaging was included), with no other explanations for the symptoms. ‘Possible’ TBM included cases that met the clinical criteria and had a diagnostic score ranging from 6 to 9 (or 6 to 11 with imaging), but no alternative diagnosis was evident.

### Exclusion criteria

The exclusion criteria for this study were as follows: ①Patients with symptoms of meningitis who did not meet the criteria for classification as ‘definite,‘ ‘probable,‘ or ‘possible’ TBM, according to the reference guidelines. ②Patients with contraindications to VPS surgery, including, but not limited to, active surgical site infections, coagulation disorders, or severe systemic diseases. ③Patients who had undergone VPS or other types of CSF shunts for hydrocephalus prior to the start of this study. ④Patients receiving concurrent treatment for other forms of tuberculosis (e.g., pulmonary tuberculosis) that could have affected the outcome of TBM-associated hydrocephalus. ⑤Patients lost to follow-up immediately after VPS surgery, making short-term and long-term evaluations impossible. ⑥Patients with incomplete medical records, including missing or inconclusive diagnostic tests or incomplete follow-up data.

### Assessment of severity and treatment effectiveness

Patients were evaluated based on Palur et al. [12] grading criteria: Grade I, characterized by normal sensation and no neurological deficits; Grade II, marked by normal sensation accompanied by neurological deficits; Grade III, presenting sensory changes while maintaining consciousness; and Grade IV, involving deep coma with or without decerebrate or decorticate posturing.

Clinical responses were assessed at two time points: at the end of the initial treatment and during the final follow-up. These responses were categorized as successful (complete remission [CR]) or partial remission [PR]) or failed (stable response, disease progression, or death).

### Follow-up and outcome evaluation

Outcomes were assessed using the Modified Rankin Scale (mRS) and the Activities of Daily Living (ADL) Scale at baseline, post-initial treatment, and during long-term follow-up to monitor for any residual neurological deficits.

### Modified rankin scale (mRS)

The mRS is commonly employed to measure disability or dependence in activities of daily living among neurologically impaired patients. It uses a 7-point scale ranging from 0 (no symptoms) to 6 (death). Lower scores indicate better recovery and less disability.

### Activities of daily living scale (ADL)

The ADL Scale assesses a patient’s ability to carry out daily self-care tasks. It evaluates a range of activities such as feeding, bathing, grooming, dressing, defecation, toileting, transferring (from bed to chair and back), and mobility. Scores range from 0 to 100, with higher scores indicating greater independence. Lower scores signify more severe dysfunction.

### Statistical analysis

All data analyses were performed using GraphPad Prism 8. For continuous variables, we examined their distribution and found that they did not follow a normal distribution. Therefore, we used the median and interquartile range (IQR) to describe these data. The median represents the central tendency of the dataset, and the IQR represents the range of data distribution. We used the Wilcoxon signed-rank test for analysis. The Kaplan-Meier method was used to plot survival curves, and differences were considered significant at a level of P < 0.05.

## Results

### General clinical characteristics

Between December 1999 and February 2023, we identified 241 patients with TBM, of whom 62 (25.7%) had hydrocephalus. Based on the inclusion and exclusion criteria, we included a total of 14 patients with TBM-associated hydrocephalus who underwent VPS. The median age of the patients was 47.5 years, and 7 (50%) were male. 3 cases had a history of pulmonary tuberculosis infection. Headache (78.6%), fever (78.6%), and altered consciousness (71.4%) were the most common symptoms. According to the Palur Grade, most cases were Grade III (8 cases, 57.1%), followed by Grade IV (5 cases, 35.7%), and only 1 case (7.1%) was Grade II. On cranial imaging, 8 cases (57.1%) had meningitis involving brain parenchyma, 1 case (7.1%) had newly developed cerebral infarction, and most cases had meningeal enhancement (8 cases, 57.1%). All patients received anti-tuberculosis treatment. The regimen consisted of isoniazid (H) at 300 mg per day, rifampicin (R) at 450 mg per day, pyrazinamide (Z) at 1,500 mg per day, and ethambutol (E) at 750 mg per day. The course of treatment lasted between 12 and 18 months. Additional treatments included steroids, dehydration measures, interventions for lowering intracranial pressure, and supportive therapies.

**Table 1:** Shunt-related complications

Case	Indication	Site	Complication	Time	Management	Outcome
1	Communicating hydrocephalus	Right	Catheter obstruction at abdominal end	4 months	Left VP shunt → obstruction again → exploration of both VP shunts → adhesion at abdominal end → relief of obstruction → cerebral herniation → conservative treatment	Death 1 year later
2	Communicating hydrocephalus	Right	Catheter obstruction at ventricular end, incision infection	5 years	Left VP shunt → obstruction again → removal of right VP shunt at the same time → right VP shunt → incision infection on the right side → removal of right VP shunt → antimicrobial therapy → right VP shunt → obstruction again → continuous lumbar cistern drainage	Death 1 year later
3	Communicating hydrocephalus	Right	Catheter obstruction at ventricular end, acute cerebral infarction	4 months	Left VP shunt → acute cerebral infarction	Death 5 years later due to multiple organ dysfunction
4	Communicating hydrocephalus	Left	Catheter infection	1 month	Removal of left VP shunt at the same time as right shunt	Paralysis due to recurrent meningitis

### Laboratory examination of CSF

**Fig. 1:**
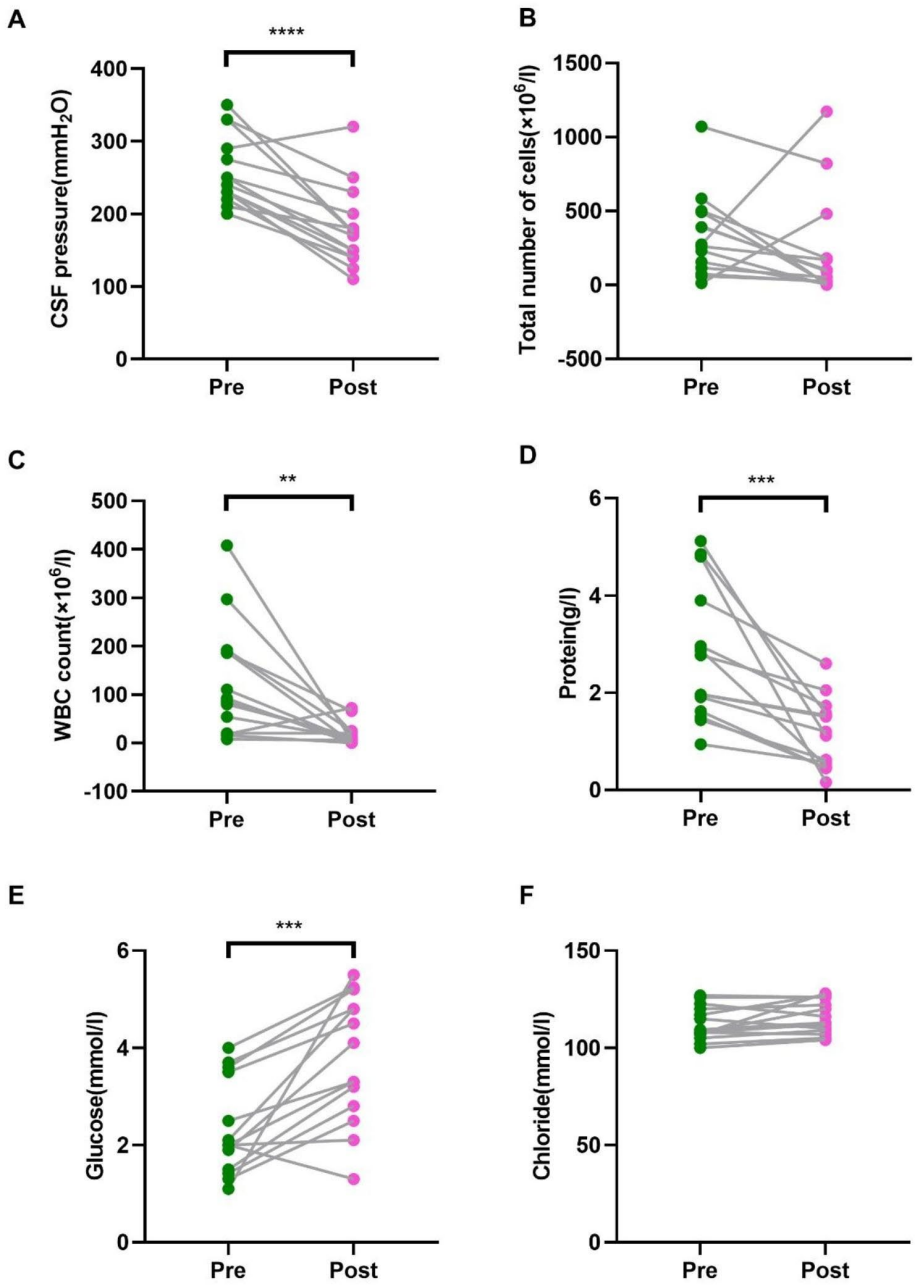
Effect of ventriculoperitoneal shunt on CSF laboratory results. **A**. CSF pressure. **B.** CSF total cell count. **C.** CSF white blood cell count. **D**. CSF protein level. **E**. CSF glucose level. **F**. CSF chloride level. ** P < 0.01, *** P < 0.001, **** P < 0.0001

All 14 patients underwent CSF examination before and after treatment. The initial lumbar puncture pressure was elevated in all patients, with a median of 245 mmH_2_O (IQR, 230.0-286.3). CSF laboratory examination revealed elevated total cell count of 267 × 10^6^/l (IQR, 125.75-468.25), elevated white blood cell count of 89 × 10^6^/l (IQR, 28.5-187.5), and elevated protein level of 2.37 g/l (IQR, 1.7–3.7), as well as decreased glucose concentration of 2 mmol/l (IQR, 1.6–3.25) and chloride level of 108.7 mmol/l (IQR, 107.3-119.3). The differences in CSF pressure, white blood cell count, protein, and glucose levels before and after shunt placement were statistically significant (P < 0.05), while the difference in total cell count and chloride level was not statistically significant (P > 0.05), as shown in Fig. 1. *Mycobacterium tuberculosis* was clearly cultured from CSF of 6 patients (42.3%).

### Initial treatment outcome

A total of 14 patients underwent VPS for TBM-associated hydrocephalus. The median duration of initial treatment was 34.5 days (IQR, 24.0-79.5). The effective response rate of initial treatment was 92.9% (13/14). 6 cases achieved CR, 7 cases achieved PR, and 1 case failed to respond. The treatment failure patient had preoperative herniation and consciousness disturbance and remained stable after the operation. There were no deaths during the initial treatment phase, and no catheter-related complications were observed. At the end of initial treatment, we evaluated mRS and ADL of all 14 patients. The median mRS score was 1 (IQR, 0-2.8), and the median ADL score was 90 (IQR, 81.3–100).

### Long-term treatment outcome

**Fig. 2:**
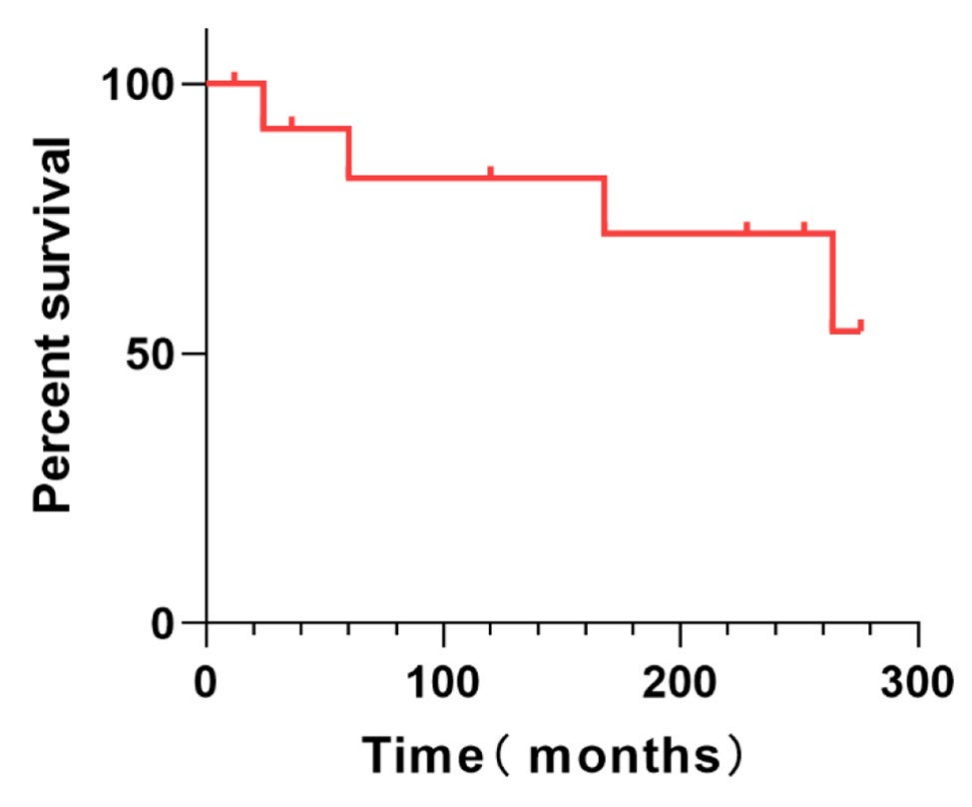
The Kaplan Meier survival curve for patients with ventriculoperitoneal shunt

A total of 14 patients completed long-term follow-up, with a median follow-up time of 168 months (IQR, 42.0-261). 4 cases (28.6%) died at the end of the last follow-up. Two died due to worsening condition after repeated catheter blockage, one due to pulmonary infection, and one due to multi-organ failure. The Kaplan-Meier survival curve is shown in Fig. 2. Long-term neurological sequelae included 4 cases of consciousness disturbance, 2 cases of limb weakness and difficulty in walking, 1 case of visual impairment, and 1 case of recent memory decline. Long-term mRS and ADL scores were evaluated in all cases, with a median mRS score of 2 (IQR, 0-5.8) and a median ADL score of 85 (IQR, 0-98.75), which were lower than those in the recent follow-up, but overall, the neurological recovery status and daily living ability were good.

### Shunt-related complications

During long-term follow-up after surgery, a total of 5 shunt-related complications were recorded, as shown in Table 1. The most common shunt-related complication was catheter obstruction (1 abdominal end and 2 ventricular ends). 1 case developed incision infection after re-shunting, 1 case developed catheter-related infection after shunting, 1 case developed acute cerebral infarction after re-shunting, and 1 case developed transient peritoneal irritation symptoms with diarrhea after shunting, which improved after symptomatic treatment. Regarding catheter obstruction complications, we further compared the impact factors of catheter obstruction in 14 patients, and the results showed that CSF protein was closely related to catheter obstruction, as shown in Table 2.

**Table 2:** Factors affecting shunt catheter obstruction

Factors	VPS for hydrocephalus		*p* -value
	Obstruction Non-obstruction		
Gender(male/female)	5/6	2/1	0.50
Age[years, M(P25,P75)]	40.0(38.0,49.0)	48.0(40.5,60.0)	0.53
GCS[points, M(P25,P75)]	9.0(8.5,9.5)	10.0(9.5,15.0)	0.15
Pressure [mmH_2_O, M(P25,P75)]	250.0(240.0,270.0)	240.0(225.0,302.5)	0.75
Total cell[×10^6^/l, M(P25,P75)]	492.0(323.5,781.0)	260.0(95.0,393.5)	0.24
WBC[×10^6^/l, M(P25,P75)]	110.0(95.0,259.0)	86.0(19.0,187.0)	0.31
Glucose[mmol/l, M(P25,P75)]	2.1(1.6,3.1)	2.0(1.7,3.0)	0.82
Chloride[mmol/l, M(P25,P75)]	117.0(113.2,121.5)	108.0(106.0,117.5)	0.18
Protein[g/l, M(P25,P75)]	4.8(3.9,5.0)	2.0(1.6,2.9)	**0.04**

## Discussion

TBM is the most lethal form of tuberculosis and poses a significant concern in developing countries. A recent meta-analysis showed that 1,250 out of 6,896 TBM patients across 21 studies died, yielding a mortality rate of 20.42% [12]. In China, the mortality rate due to TBM varies widely, ranging from 2.38 to 36.0%. This variation can be attributed to factors such as regional differences, the quality of healthcare facilities, and co-existing HIV infections [13, 14]. One primary contributor to the elevated mortality rate is TBM-induced hydrocephalus, which leads to acute intracranial hypertension. Therefore, effectively managing intracranial pressure is critical for ensuring adequate cerebral blood flow while administering anti-tuberculosis treatment [1].

While Ventriculoperitoneal shunting (VPS) has proven effective in promptly alleviating intracranial hypertension caused by TBM, questions remain about its long-term safety and efficacy, particularly concerning catheter-related complications. In this context, our study is the first to conduct a comprehensive, long-term follow-up on TBM patients, ranging from one to 23 years, with the aim of holistically assessing the long-term efficacy and safety of VPS in TBM treatment.

To accurately assess the baseline condition of patients, we initially carried out an exhaustive evaluation of VPS’s efficacy and safety during the initial phase of treatment. Notably, we observed a significant alleviation of intracranial pressure post-shunt surgery, a finding that aligns closely with previous research [15]. The use of a pressure-adjustable shunt valve enabled dynamic regulation of intracranial pressure, providing unique advantages in managing this critical parameter [16]. Following VPS surgery, we observed a significant improvement in the biochemical markers of cerebrospinal fluid (CSF) in patients. This improvement is likely attributable to the drainage of *Mycobacterium tuberculosis* into the peritoneal cavity via the VPS shunt, thereby enhancing CSF circulation and augmenting the therapeutic effectiveness against TBM. These observations are consistent with our previous findings on the efficacy of VPS in treating cryptococcal meningitis [17].

In this investigation, we observed no immediate postoperative deaths during hospitalization following VPS surgery, suggesting an extraordinarily high treatment response rate. This adds further evidence to substantiate VPS as a viable solution for mitigating intracranial hypertension induced by TBM. However, it is crucial to note that during the initial phase of treatment, 4 cases (or 28.6% of our sample) developed neurological sequelae. This outcome may correlate with the predominance of Grade III-IV patients within our study cohort. Other research also indicates that Grade III-IV patients exhibit a substantially higher rate of disability compared to those in Grades I-II [18]. Subsequently, we conducted a thorough long-term evaluation of VPS’s efficacy and safety. During the extended follow-up period, we regrettably noted that a total of four patients passed away. Two succumbed due to the rapid worsening of their conditions, triggered by repeated catheter obstructions, while the remaining two deaths were attributed to other causes. Additionally, eight patients (or 57.1% of the sample) continued to suffer from persistent neurological sequelae. Significantly, for the first time, we conducted a comparative analysis of neurological scores (mRS) and quality of life scores (ADL) from the initial treatment stage through long-term follow-up in TBM patients. Although both mRS and ADL scores decreased compared with the initial treatment period, with the median mRS score decreasing from 1 to 2 in the initial period and the median ADL score decreasing from 90 to 85 during the long-term follow-up period, these scores still remain within a relatively encouraging range. Further research is essential to corroborate the long-term effects on neurological function and daily quality of life.

TBM is associated with a higher complication rate after VPS compared to other types of secondary hydrocephalus [9]. A retrospective study involving 217 patients at stages II to III of TBM with hydrocephalus found that complications occurred in as many as 32.3% of cases. These primarily included shunt obstructions and infections [19]. Echoing these findings, our study also highlighted a postoperative complication rate of 28.6%, with shunt obstruction being the most prevalent issue. This phenomenon is likely attributable to elevated protein concentrations in the CSF. This hypothesis is supported by Kamat et al. [20], who demonstrated a significant correlation between increased CSF protein levels and VPS obstructions in TBM patients. Furthermore, Ambekar et al. [21] reported a strong association between CSF protein levels and shunt malfunctions, although they found no direct correlation with CSF cell counts or glucose levels. Their data revealed that patients with CSF protein concentrations exceeding 200 mg/dL faced a risk of shunt dysfunction four times greater than those with concentrations below 100 mg/dL [21]. It is noteworthy that patients may encounter recurrent shunt obstructions within the first year post-surgery due to elevated CSF protein concentrations, contributing to suboptimal treatment outcomes [22, 23]. In situations presenting a high risk of VPS tube blockage, endoscopic third ventriculostomy (ETV) emerges as a viable alternative, avoiding most VPS-associated complications [24]. However, it is imperative to consider that acute VPS treatment for TBM could potentially facilitate the intraperitoneal spread of tuberculosis. Bhople et al. [25] documented a pediatric case in which *Mycobacterium tuberculosis* spread into the peritoneal cavity along the shunt, forming a tuberculous granuloma. However, evidence supporting this claim remains limited. In our study, we observed a single instance of transient irritant peritoneal syndrome accompanied by diarrhea, which resolved spontaneously within a week.

This study is subject to several significant limitations that warrant consideration. First, the study employs a retrospective design, making it susceptible to both selection and information biases. Second, the limited sample size could potentially undermine the generalizability and statistical robustness of our findings. Lastly, although our follow-up period spans an extensive range of 1 to 23 years, the duration of this follow-up varies considerably among individual patients, which could compromise the reliability of our long-term outcomes. Therefore, future research involving larger sample sizes is imperative to enhance the robustness and reliability of these findings.

In conclusion, VPS serves as an efficacious and well-tolerated therapeutic intervention for hydrocephalus associated with TBM. The procedure offers a robust means of mitigating acute intracranial hypertension, thereby enhancing patient prognosis. Nonetheless, given the substantial rate of complications, diligent monitoring and proactive management are imperative for optimal treatment outcomes.

## Data Availability

The datasets generated and analyzed during the current study are not publicly available due to privacy or ethical restrictions but are available from the corresponding author on reasonable request.
